# Arthritis as presenting manifestation of acute lymphoblastic leukemia in children

**DOI:** 10.1186/1546-0096-12-S1-P60

**Published:** 2014-09-17

**Authors:** Ninna Brix, Troels Herlin, Henrik Hasle, Steen Rosthøj

**Affiliations:** 1 pediatric department, Skejby hospital, Aarhus N, Denmark; 2 pediatric department, aalborg hospital, aalborg, Denmark

## Introduction

At disease onset children with acute lymphoblastic leukemia (ALL) may present with arthralgia (join pain) or even signs of arthritis with joint swelling, redness, and restriction of motion. This might cause misdiagnosis and thereby lead to prolonged diagnostic delay. The present study aimed to identify ALL children with joint involvement and to compare their characteristics and outcome with ALL children without joint involvement.

## Objectives

Case records of 286 children diagnosed with ALL between 1992 and 2013 were reviewed and analyzed in this retrospective, descriptive study.

## Methods

Data analysis was mainly descriptive and selected differences in frequencies were tested for statistical significance using Fisher’s exact test with two-sided tests and 5% level of significance. The overall and event free survival were determined using Kaplan-Meier plotting.

## Results

Eighteen percent of the children with ALL presented with localized joint pain and half (9%) had objective signs of arthritis. The mean number of joints involved was 2.5, most frequently presenting as asymmetric oligoarthritis. The suspected misdiagnosis were: Reactive arthritis: 19/53, osteomyelitis: 9/53 and juvenile idiopathic arthritis: 8/53. Children with joint involvement had less objective signs of leukemia: Cytopenia was absent in 24% (vs. 8%, p=0.001), 50% had less than two cell lines affected (vs. 21%, p=0.0005), 44% had no organomegaly (vs. 29%, p=0.05). Median diagnostic delay was 4 weeks vs 2 weeks. The 5-year event-free survival was better for children with joint involvement compared to those without (94% vs. 87%, p=0.049), as well as the 5-year overall survival (96% vs. 83%, p=0.044).

## Conclusion

Acute lymphoblastic leukemia with joint involvement is a frequent finding (18%). At presentation cytopenia and hepatosplenomegaly are rarely seen in ALL with joint involvement. The risk of an initial rheumatic misdiagnosis was significant. The overall and event-free survival were superior compared to the children without joint involvement

## Disclosure of interest

None declared.

